# Various inflammatory phenotypes in V200M NLRP3 carriers

**DOI:** 10.1186/1546-0096-13-S1-P13

**Published:** 2015-09-28

**Authors:** A Kozlova, V Bobrynina, T Varlamova, A Maschan, A Shcherbina

**Affiliations:** 1 Federal Research and Clinical Center of Pediatric Hematology, Oncology and Immunology, immunology, Moscow, Russian Federation

## Background

Cryopyrin-associated periodic syndrome (CAPS) is a very rare auto-inflammatory syndrome. CAPS is caused by mutations of the NLRP3 gene that encodes crypopyrin protein that is a part of inflammasome complex. CAPS patients can present with different phenotypes of the disease - Familial cold urticaria, Muckle-Wells syndrome, and the most severe phenotype - neonatal onset multisystem inflammatory disease (NOMID). CAPS patients experience symptoms of systemic inflammation, intense fatigue and have poor quality of life. In the most severe forms, they may develop serious organ damage such as visual and hearing impairment, arthritis, neurological deterioration and renal insufficiency. However, V200M mutation has been associated with mild inflammatory phenotype and its pathogenic role is even questioned by some. We report two families of V200M mutation carriers, with variable inflammatory phenotypes. In all cases mutations of MEFV, MVK, TNFRSF1A genes were excluded.

## Family 1

Index case is a 1,5 years old female, that has been suffering from symptoms similar to Muckle-Wells syndrome: weekly episodes of fever and urticarial rash since infancy, accompanied by high laboratory inflammatory parameters (elevated ESR, CRP, hypergammaglobulinemia). Focal and diffuse changes were found on brain MRI, mild developmental delay was noted. Cerebrospinal fluid, audiogram were normal. She was started on IL1 inhibitor therapy (anakinra) with complete resolution of her symptoms. Sister, mother and grandmother of girl have the same mutation. The sister and mother have no symptoms of the disease. Yet, grandmother started having symptoms of recurrent fever, arthritis since the age of 3 years, and currently, at the age of 60 years, has a significant a hearing loss.

## Family 2

The index case, 4 year old female, has been suffering from 2 years of age from monthly episodes that could be classified as PFAPA (periodic fever-pharyngitis-polyadenitis- aphtous stomatitis): 1-2 days long episodes of fever, pharyngitis, stomatitis and high laboratory activity. Brain MRI, cerebrospinal fluid, audiogram, vision were normal. Girl's father carries the same mutation, but he has no clinical symptoms of PFAPA or CAPS

## Conclusions

The NLRP3 V200M variant carriers show variable expressivity of the disease, the pathogenic role of this mutation and indications for therapy require further investigation. It will be interesting to screen typical PFAPA patients for V200M carrier status.

## Consent to publish

Written informed consent for publication of their clinical details was obtained from the patient/parent/guardian/relative of the patient.

